# Construction and validation of nursing diagnoses for people in palliative care^1^

**DOI:** 10.1590/1518-8345.1862.2914

**Published:** 2017-08-03

**Authors:** Rudval Souza da Silva, Álvaro Pereira, Maria Miriam Lima da Nóbrega, Fernanda Carneiro Mussi

**Affiliations:** 2 PhD, Adjunct Professor, Universidade do Estado da Bahia, Senhor do Bonfim, BA, Brazil.; 3 PhD, Associate Professor, Universidade Federal da Bahia, Salvador, BA, Brazil.; 4 PhD, Full Professor, Universidade Federal da Paraíba, João Pessoa, PB, Brazil.

**Keywords:** Palliative Care, Nursing Diagnosis, Classification, Terminology

## Abstract

**Objective:**

to construct and validate nursing diagnoses for people in palliative care based on the Dignity-Conserving Care Model and the International Classification for Nursing Practice.

**Method:**

a two-stage methodological study: 1) construction of the database of clinically and culturally relevant terms for the nursing care for people in palliative care and 2) construction of nursing diagnoses from the database of terms, based on the guidelines of the International Council of Nurses.

**Results:**

the 262 terms validated constituted a database of terms from which 56 nursing diagnoses were developed. Of these, 33 were validated by a group of 26 experts, and classified in the three categories of the Dignity-Conserving Care Model: illness-related concerns (21); dignity-conserving repertoire (9); and social dignity inventory (3).

**Conclusion:**

of the 33 validated diagnoses, 18 of them could be included in the update of the Catalog of the International Classification for Nursing Practice - palliative care for a dignified death. The study contributes to support the clinical reasoning and decision making of the nurse.

## Introduction

Care for the person, in the process of dying and facing death, is part of the experience of the health team, especially of Nursing professionals who are continuously present and directly provide the majority of the care to the person. They offer care when healing is no longer a possibility and even provide care for the postmortem body and during mourning^1^.

There is a clear need for health professionals to seek care for the promotion, prevention of injuries and recovery of health, as well as to valorize a dignified death, assuming that death should not be an enemy to be overcome, but a natural event that is integral to life^2^. With this in mind, every day the philosophical principles regarding palliative care have gained strength and space in the care settings.

Palliative care is defined by the World Health Organization^3^ as an approach to care that seeks to improve the quality of life of individuals and their family members when faced with problems arising from the illness and from the risk to life, through prevention, minimization and relief from the suffering. This can be achieved by early identification, assessment and treatment of pain and other physical, psychosocial and spiritual problems.

Palliative care is, at the same time, a guiding philosophy and guideline for actions to be undertaken by a multidisciplinary health team, structured in an interdisciplinary care system^3^. Its principles can be applied to all patients, in different age groups, and their families, with emphasis on care for the preservation of dignity, and having the relief from suffering as the focus of the care^4^.

The number of palliative care programs has been increasing rapidly in recent years due to the greater amount of people with chronic and life-threatening illnesses, associated with greater involvement of families in the decisions about the end-of-life care for their loved ones^5^.

The participation of the nurse in the palliative care context is essential, considering that this care is performed in an area of ​​health intervention, in which the role of the nurse represents the link between the patient, the family and the other members of the team, with this professional having a greater opportunity to perform care practices, due to spending much of the time with the patient and family^1^.

Considering the above, it is relevant to undertake studies regarding care for the person in palliative care, from the Systematization of Nursing Care as a tool for work organization and the application of the Nursing Process, highlighting the relevance of terminologies inherent to the elements of the practice for the documentation of the care process, in favor of a standardized language.

The International Classification for Nursing Practice (ICNP^®^) emerges as a unifying framework for language. It provides terminology to support the critical thinking of nurses in care planning, with a view to facilitating communication, documentation and greater visibility to nursing actions, as well as contributing to the development of electronic records and the advancement of knowledge^6^ ^-^ ^7^.

There are different theories and conceptual models that seek to explore the different theoretical models related to dignity, which have been developed in different contexts of clinical practice^8^. For this study, the Dignity-Conserving Care Model (DCCM)^9^ was chosen because it is a reference in the context of palliative care, and was already used in the first edition of the ICNP^®^ Catalog - Palliative Care for Dignified Dying^10^, as well as being the theoretical model that specifically defines “dying with dignity”^11^. The model is composed of the following main categories: illness-related concerns; dignity-conserving repertoire and social dignity inventory^9^.

This model and the Nursing Diagnoses (NDs) aim to provide a structure for nurses to plan an individualized approach directed toward conserving the dignity of the person, in the process of dying and facing death.

The present study aimed to contribute to the expansion, consolidation and updating of the existing Catalog^10^, published in 2009 by the International Council of Nurses (ICN), and developed from studies conducted in Ethiopia, Kenya, India, the Philippines and the USA^11^ ^-^ ^13^.

The results of this study contribute to filling gaps related to the relevant NDs in the context of palliative care, such as the diagnosis of “preserved dignity”, which is not part of the ICNP 2011, and is not included in the Catalog^10^. With this, it will be possible to direct interventions in this field of care, in health and nursing, as well as to provide evidence for the practice of the nurse in the context of palliative care, considering the lack of studies on NDs for palliative care patients^14^.

Therefore, the present study aimed to construct and validate NDs for people in palliative care, based on the DCCM^9^ and the ICNP^®^.

## Method

This methodological study used the recommendations of the ICN for the development of terminological subsets^15^, based on the Database of Terms (DT) constructed in the first stage of this study^16^ ^)^ and the reference model of the NDs of the ISO 18.104: 2014 Standard^17^.

The research project was evaluated by the Research Ethics Committee of EEUFBA, in compliance with the ethical aspects recommended in Resolution No. 466/12 of the Ministry of Health, and obtained approval under authorization No. 353.005.

The study was developed through the following steps: 1) construction of the database of clinically and culturally relevant terms for the nursing practice with people in palliative care. In this step, a descriptive-documentary study was carried out^16^, which resulted in a database of 262 terms, which subsidized the next step. 2) construction of the NDs from the DT^16^, based on the ICN guidelines. This step composed the object of this publication.

The construction of the NDs was operationalized in four different moments: 1) construction of the NDs and their operational definitions; 2) content validation by experts selected according to the Fehring’s modified criteria^18^; 3) application of the Content Validity Index (CVI), being adequate when ≥0.80 and 4) cross-mapping between validated NDs and those in the Catalog^10^.

Following the methodological steps for the construction of the NDs, the diagnoses were initially constructed based on the reference model^17^, which determines that a term of the ICNP^®^ Seven Axes Model inherent to the focus axis and another to the judgment axis should be mandatorily included. The inclusion of additional terms from the other axes is optional. The theoretical framework of the DCCM^9^ was also taken into consideration.

For the development of the operational definitions, the following methodological strategies were used: review of the literature, mapping of the meaning of the concept and affirming the operational definition^19^. For these definitions, the palliative care area of clinical specialty​​ was considered and, for each one, the specific characteristics to guide its identification were established.

After the development of the NDs and their operational definitions, the resulting product was submitted to the content validation process by selected experts, according to Fehring’s modified criteria^18^. In this study, the adaptation performed was related to flexibility in the participation of nurses without the Master’s degree, provided they had a specialization course or residency with a focus on palliative care. Studies highlight that Fehring’s criteria^18^ are still the most used, mainly through adaptations^20^.

The sample universe consisted of 75 Brazilian nurses who had a minimum of a Master’s degree and/or specialization/residency in palliative care, who worked with NDs and palliative care in the area of care, teaching or research. Subjects with Fehring’s criteria^18^ scores lower than five were excluded from the selection of experts. Thus, of the 283 experts recruited, after applying the criteria, the intentional sample was obtained. The selection of the subjects was carried out through an active search of expert professionals from the aforementioned areas, in the Plataforma Lattes, of the National Council for Scientific and Technological Development - CNPq (Curriculum Lattes and Directory of Research Groups).

Initially, an e-mail was sent to the experts, with an invitation letter, the consent form, orientations about the study and the research instrument comprising a relationship with 56 NDs. The instrument presented the NDs, followed by the operational definitions and a five-point Likert type scale (1 = not relevant, 2 = slightly relevant, 3 = fairly relevant, 4 = relevant, 5 = very relevant) for assessing the relevance of each definition.

Those that agreed to participate in the study returned the material by e-mail, after a period of approximately four months (January to April 2014), after repeated submissions of the invitation, obtaining a sample of 26 (34.7%) adequately completed instruments. Four of the experts invited justified not participating due to having experience in palliative care, but not with the Classification System - ICNP^®^, and vice versa.

For the analysis of the responses of the experts, the CVI was applied, the formula of which consists of:



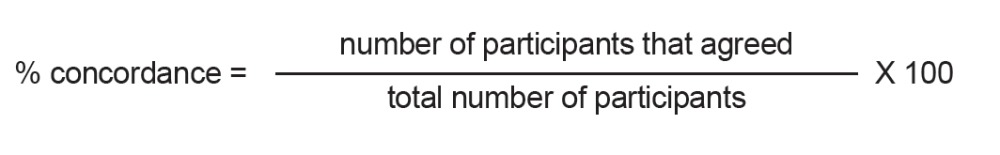



The ND and its respective operational definition were considered relevant when the CVI≥0.80. This score was adopted as the coefficient of reliability, considering that the literature recognizes this as a standard cut-off point as a weighted measurement tool^21^.

Next, the validated NDs (CVI≥0.80) were submitted to the cross-mapping technique^22^, with these being crossed with those included in the Catalog^10^, to identify whether or not they were included. This process took place by typing the NDs into a Microsoft Office Excel^®^ 2010 worksheet, then importing it into the Microsoft Office Access^®^ 2010 program, with the cross-mapping technique being used, which made it possible to compare the ND products of this study with those of the Catalog^10^.

Finally, the categorization stage occurred, according to the DCCM^9^, when the NDs were classified in accordance with the categories: illness-related concerns, dignity-conserving repertoire and social dignity inventory. The categorization was based on the analysis performed by the principal researcher and then went through a process of evaluation and validation by a group of three nurse practitioners with experience in palliative care.

## Results

In the first stage of the study, the terms were identified from the interviews with professionals of the nursing team, which gave a total of 432 terms^16^. Of these, after the process of identification of the meanings and similarities and the treatment of standardization, 170 (39.3%) were excluded, considered junk terminology, resulting in 262 (60.7%) terms, which composed the DT to support the second stage of this study.

It should be mentioned that of the 262 terms that made up DT, 167 (63.7%) were already included in ICNP^®^ 2011, and 95 (36.3%) were classified as non-constant^16^.

Using the DT, and directed by the ISO 18.104:2014 standard - reference terminology model for Nursing - and the DCCM, 56 positive or negative NDs were constructed, including diagnoses and well-being and their respective operational definitions.

After the evaluation by the experts, of the 56 NDs elaborated, 33 (58.9%) obtained IVC≥0.80 (Table 1).

**Table 1:** Distribution of the Nursing Diagnoses with Content Validity Index ≥0.80. Salvador, BA, Brazil, 2014

**Nursing Diagnoses**	CVI*
**Adaptation to changes impaired**	**0.80**
**Adherence to the therapeutic regimen**	**0.90**
**Anxiety related to death**	**0.90**
**Impaired psycho-spiritual aspect**	**0.80**
**Impaired attitude of coping with the death and dying process**	**0.80**
**Impaired communication**	**0.80**
**Discomfort**	**0.90**
**Hopelessness**	**0.90**
**Despair**	**0.90**
**Conserved dignity**	**0.90**
**Dyspnea (specify degree)**	**0.90**
**Pain (specify intensity)**	**0.90**
**Edema (specify degree)**	**0.80**
**Impaired emotional state**	**0.80**
**Expectation of hope achieved**	**0.80**
**Fatigue**	**0.80**
**Lack of family support**	**0.80**
**Wound (specify location)**	**0.80**
**Hypertension**	**0.80**
**Hypothermia**	**0.80**
**Hypoxia**	**0.80**
**Nausea**	**0.80**
**Improved orientation**	**0.80**
**Impaired orientation**	**0.80**
**Decision-making process impaired**	**0.80**
**Impaired patient/caregiver relationship**	**0.80**
**Impaired respiration**	**0.80**
**Risk of spiritual distress**	**0.80**
**Risk of cachexia**	**0.80**
**Risk of interruption of self-care**	**0.80**
**Impaired sleep**	**0.80**
**Chronic sadness**	**0.80**
**Will to live present**	**0.80**

*Content Validity Index

**Figure 1:**
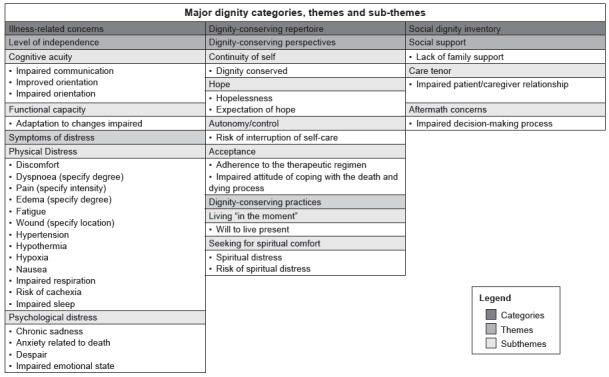
Distribution of NDs in the DCCM categories^9^. Salvador, BA, Brazil, 2014

The 33 NDs were submitted to the cross-mapping technique and then categorized according to the DCCM^9^ ^)^ (Figure 1). When they were crossed with the Catalog^10^ it was evident that only 8 of the 33 DEs were in the catalog: spiritual distress, discomfort, hopelessness, pain, fatigue, nausea, impaired respiration and impaired sleep. It should be mentioned that 15 NDs presented in Table 1 are not included in the ICNP^®^ 2011.

Figure 1 shows that 21 diagnoses were classified in the illness-related concerns category, 9 in the dignity-conserving repertoire category and 3 in the social dignity inventory category.

## Discussion

The ICN considers palliative care a priority^15^ for the development of ICNP^®^ Catalogs and, from this perspective, recognizes the phenomenon of “dying with dignity” as inherent in the nursing care, as well as adopting the DCCM^9^ ^)^ as a reference for structuring the Catalog^10^. This enables nurses to plan the nursing care taking into account the preservation of human dignity^23^.

The theoretical model adopted in the study specifies three main categories related to the dignity of the person in palliative care. The first is illness-related concerns, which deals with the management of the needs inherent to the control of physical and psychological symptoms, considering that the control of pain at any time, especially in end-of-life care, is critical to the success of improving care for those who are dying. The second, the dignity-conserving repertoire, considers that the human response to disease is not only determined by the disease itself, but by the totality of the person that is in a condition of illness. Each person is considered to have a specific psychological profile, as well as a spiritual perspective that makes it possible to shape their world view and their responses to opportunities and crises. Finally, the social dignity inventory category refers to the social and/or dynamic issues of relationships that increase or diminish the sense of dignity of each person^9^.

Of the terms identified as not included in the ICNP^®^, those belonging to the focus axis are highlighted, as they represent the focus of attention for the systematization of nursing care. Of the 95 non-constant terms, 33 (34.7%) were classified in this axis^16^, and 62 in the other 6 axes of the Seven Axes Model of the ICNP^®^.

Among these 33 terms, those inherent to the dignity of the person in palliative care were evidenced, such as: psychological support, moral support, psycho-spiritual aspect, good death, humanization, respect, responsibility and singularity, among others. The word dignity means to be worthy of honor, respect or esteem^9^. Its concept is considered to be one of the most important professional values, being of great relevance to Nursing, due to the human nature of its professional practice. Hence, caring, considering dignity-conserving care, means respecting the human individuality and treating each person as a unique being, thus becoming a basic human need and an important aspect in nursing care^24^. Therefore, it is necessary to consider the aspects identified in the study as the focus of the nursing care, among them the singularity, respect and moral and psychological support.

Based on the DCCM^9^, the focus of care, from these 33 terms, is directed toward the two main categories in the context of palliative care: the dignity-conserving repertoire and the social dignity inventory. However, these two categories grouped a smaller number of diagnoses, according to the Catalog^10^ and this study.

Dignity is conceived from intrinsic and extrinsic components^9^, the latter being influenced by environmental and cultural circumstances, which tend to impact on the dignity of each person. Therefore, each individual, faced with their condition of illness, responds differently to coping with the situation.

In a randomized clinical trial^25^, developed in New York City with patients in palliative care, the “Dignity Therapy” intervention, a brief psychotherapy, was provided, offering patients the opportunity to talk about what matters the most for them faced with the death and dying process. The intervention was applied by a team consisting of a nurse, a psychologist and a psychiatrist, and evidenced a positive response from the patients, especially in improving the spiritual well-being and the way the family saw them. This shows the influence of the intrinsic and extrinsic components, which influence the dignity of the person.

The “illness-related concerns” and “dignity-conserving repertoire” categories are interrelated and refer to the physical, psychological, and existential factors internalized in each person’s life experience and how they influence the sense of their dignity. The “social dignity inventory” conceptually overlaps the extrinsic components of dignity, and refers to how other people and environmental circumstances can influence the sense of dignity of a person^9^.

This shows how necessary it is to think about the importance of emphasizing nursing care planning, focusing on active listening and the establishment of NDs that consider the individuality of each person, with respect to their autonomy in the decision-making process. In this moment, the nurse should be emphatic in the use of clinical and therapeutic reasoning, allowing greater accuracy in the selection of the NDs directing the focus of care toward the nursing actions.

Of the 33 validated diagnoses, 10 did not appear in the Catalog^10^ and 15 did not appear in the ICNP 2011, highlighting gaps in phenomena related to dignified death. This included “impaired communication”, with effective communication considered a fundamental element in palliative care for the development of the therapeutic relationship between patients and nurses^26^, making the relief of anxiety, control of the situation and promotion of quality of life possible.

Other NDs that did not exist in the Catalog^10^ were: despair, preserved dignity, impaired emotional state, impaired orientation, risk of cachexia, chronic sadness and the will to live present; Only “chronic sadness” was included in the ICNP 2011. All of them presented adherence to the categories of the theoretical model and are applicable in the context of palliative care. In this sense, the results of this study provide contributions, based on scientific evidence, that reinforce the relevance of updating the existing catalog.

Another point that deserves attention is the fact that an ND in the subtheme “generativity/legacy”, of the dignity-conserving repertoire category of the DCCM was not identified in the study, nor is it listed in the Catalog^10^. For the care contents theme, of the social dignity inventory category, there is no diagnosis in the Catalog^10^, however, in this study the “impaired patient/caregiver relationship” was evidenced.

The care content is a theme that correlates with the attitude that others (family, health professional or caregiver) demonstrate when interacting with the patient. In a concept analysis study regarding the continuity of care at the end of life, focusing on the perspective of the patient, it was discovered that continuity of care at the end of life is a dynamic process and depends precisely on the interaction between patients, family members and providers, which is closely linked with the perception of the time of the patient in his/her dying process. It was evidenced in the study that the nurse can benefit from a deeper understanding of the experience of the patient regarding factors that hinder the care process, such as impaired communication and difficulties in interpersonal relationship, as well as those related to planning the care with attention to the relief of symptoms, the self-image, and the recognition of the proximity with death^27^.

The “impaired adaptation to change” ND was classified in the “functional capacity” subtheme, in the illness-related concerns category, considering its operational definition and the concepts of categories and subcategories of the theoretical model. However, in the Catalog, the “impaired adaptation” ND is classified in the theme “maintaining normality”, of the “dignity-conserving repertoire” category, for which no ND was identified in the present study.

The classification of “impaired adaptation to change” in the “functional capacity” subtheme was guided by its definition in the theoretical model^9^: which refers to the ability to perform activities of daily living such as shopping, bathing and preparing meals, among others.

The “preserved love” ND did not achieve the defined CVI, however, there were pertinent suggestions regarding its modification to “positive self-esteem”, which is related to the profile of the adopted model and is already an ND contemplated in the Catalog^10^ in the main category of “dignity-conserving repertoire” - sub-theme “maintenance of pride”. It should be noted that for this subtheme, no diagnosis was evidenced in the present study.

The NDs “risk of injury”, “risk of emotional problem”, “risk of sadness” and “risk of pressure ulcer” did not achieve CVI≥0.80. The experts did not justify the non-relevance of these NDs, nor offer suggestions for improvements. The index of NDs of risk in the classification systems is still very low.

The question remains regarding why the “risk of sadness” ND did not achieve the desirable CVI, while “chronic sadness”, a condition secondary to the diagnosis of potentiality, did.

The results of this study particularly contribute to the updating of the existing catalog, as well as highlight scientific evidence that can be applied in the clinical practice and even be tested through the clinical validity of the NDs and their relationships in the respective categories and subcategories of the DCCM. In addition, the elements of the nursing practice for the promotion of a dignified death were explored from the perspectives of the individuals and their family members.

## Conclusion

The 33 NDs validated in this study, and classified in different categories of the DCCM, express a common language for Nursing, aiming to guide the systematized planning of nursing care. They also contribute to the implementation of the Nursing Process and the use of the ICNP^®^ as an international nursing language system, which aims to support the planning and management of palliative care by the nursing team, in order to promote a dignified death.

A limitation of the present study was the fact that the data were obtained from a database where the information does not allow generalizations, as it demonstrates the profile of a given reality. Furthermore the NDs developed were not submitted to clinical validation. Therefore, other studies should be conducted in order to identify new terms that can be added to them, considering the reality of other empirical fields, as well as to enable the clinical validation of NDs and to verify their applicability in the context of palliative care, whether in the hospital or home context.
